# Possible mechanisms of kidney repair

**DOI:** 10.1186/1755-1536-2-3

**Published:** 2009-06-26

**Authors:** Paola Romagnani, Raghu Kalluri

**Affiliations:** 1 Excellence Centre for Research, Transfer and High Education DENOthe, University of Florence, Florence, Italy; 2 Beth Israel Deaconess Medical Center, Boston, Massachusetts, USA; 3 Department of Medicine, Harvard Medical School, Division of Matrix Biology, Department of Medicine Beth Israel Deaconess Medical Center, Boston, Massachusetts, USA

## Abstract

In most adult epithelia the process of replacing damaged or dead cells is maintained through the presence of stem/progenitor cells, which allow epithelial tissues to be repaired following injury. Existing evidence strongly supports the presence of stem cells in the adult kidney. Indeed, recent findings provide evidence in favour of a role for intrinsic renal cells and against a physiological role for bone marrow-derived stem cells in the regeneration of renal epithelial cells. In addition, recent studies have identified a subset of CD24^+^CD133^+ ^renal progenitors within the Bowman's capsule of adult human kidney, which provides regenerative potential for injured renal epithelial cells. Intriguingly, CD24^+^CD133^+ ^renal progenitors also represent common progenitors of tubular cells and podocytes during renal development. Chronic injury causes dysfunction of the tubular epithelial cells, which triggers the release of fibrogenic cytokines and recruitment of inflammatory cells to injured kidneys. The rapid interposition of scar tissue probably confers a survival advantage by preventing infectious microorganisms from invading the wound, but prevents subsequent tissue regeneration. However, the existence of renal epithelial progenitors in the kidney suggests a possible explanation for the regression of renal lesions which has been observed in experimental animals and even in humans. Thus, manipulation of the wound repair process in order to shift it towards regeneration will probably require the ability to slow the rapid fibrotic response so that renal progenitor cells can allow tissue regeneration rather than scar formation.

## Background

Most epithelia need to constantly replace damaged or dead cells throughout life. The process of continual cell replacement is critical for the maintenance of adult tissues and is typically maintained through the presence of stem cells. Stem cells are functionally defined by their ability to self-renew and to differentiate into the cell lineages of their tissue of origin [1]. Once activated, epithelial stem cells can generate proliferating progeny, which are often referred to as transiently amplifying cells. In their normal environment, transiently amplifying cells will divide actively for a restricted period of time, expanding the cellular pool that will then differentiate along a particular cell lineage to make the tissue. The physiological replacement of cells varies substantially among different epithelia. The epithelium of the intestine completely self-renews within around 5 days. By contrast, interfollicular epidermis takes approximately 4 weeks to renew, whereas the lung epithelium can take as long as 6 months to be replaced. In addition some epithelia, such as hair follicles, present a cyclic mode of cell replacement [1]. Similarly, the mammary gland proceeds through cycles of growth and degeneration during and following pregnancy [1]. In addition, stem cells are critically involved in regeneration upon wounding. Unless the epithelial stem/progenitor cells are permanently damaged, most epithelia are able to repair their tissues following injury [1]; when epithelial stem cells are depleted, fibrotic responses occur [1].

### Do renal stem/progenitor cells exist in the adult kidney?

The understanding of kidney repair is still in its infancy despite the rapid advances made in recent years. The kidney is one of the few organs that undergo mesenchymal-epithelial transition during development [2]. Moreover, structures present in the adult kidney arise from reciprocal interactions between two discrete embryonic appendages, namely the ureteric bud (UB) and metanephric mesenchyme (MM) [2]. The adult kidney contains more than 24 mature cell types arranged in distinct vascular, interstitial, glomerular and tubular compartments [2]. This unique organogenesis and structural complexity of the adult kidney has presented many challenges to the identification and characterization of kidney stem cells [2,3]. Attempts to identify adult kidney stem cells were made on the basis of the broad principles of stem cell biology, such as prolonged cell-cycling time (label-retaining cells), ability to extrude Hoechst dye (side population cells), by restrictive cell culture conditions, or by using markers expressed by other stem cells or developing kidney [3]. Existing evidence strongly supports the presence of stem cells in the adult kidney. Indeed, remission of disease and regression of renal lesions have been observed in experimental animals and even in humans [4]. Identification and knowledge of renal stem cell biology might help to unlock latent regenerative pathways in human kidney, which would have the potential to change medical practice as much as the introduction of dialysis did in the twentieth century.

The origin of the cells that replace injured tubular epithelia is not known [3,5], although several lines of evidence suggest an intrarenal source [6,7]. Recently, putative adult kidney stem cells have been isolated, with some evidence indicating that they may enable epithelial repair after injury [8-18]. Several studies have suggested the existence of an interstitial renal stem cell. One way of looking for stem cells in solid organs was a pulse of bromodeoxyuridine (BrdU) followed by a long chase period. The quiescent stem cells, which do not divide, maintain the high levels of BrdU deposited in their genomes, whereas the dividing, more differentiated stem cells steadily dilute the BrdU incorporated into their genomes as they proliferate. Maeshima *et al*. [12,13] identified BrdU-labelled cells, which they termed renal progenitor-like tubular cells, in the renal tubules. Oliver *et al*. [14] identified a population of BrdU-label-retaining cells within the interstitium of renal papilla in the rat kidney. However, the use of BrdU labelling does not seem to be a specific method for identification of stem cells [16].

Other studies have identified a rare population of adult interstitial cells in rat or human kidney [8,9], and such cells have been proposed to engraft into tubules of either developing or injured kidney tissue [8,9], suggesting that extratubular cells can traverse the basement membrane and contribute to epithelium [14]. Although intriguing, this hypothesis has recently been questioned by a study by Humphreys *et al*. who have developed a method for distinguishing the source of kidney tubular regeneration based on studies in transgenic rodents for the homeodomain transcriptional regulator Six2 [18]. In this model, the Six2 promoter drives a fusion protein of green fluorescent protein (GFP) and Cre recombinase, which is expressed transiently in renal epithelial precursors during the developmental period of active nephrogenesis [18]. GFPCre expression is not present in the adult, and this expression is not observed after injury. When Six2-GFPCre mice are crossed with a floxed STOP reporter strain, Cre-dependent removal of the stop sequence in progeny leads to constitutive and heritable expression of a marker gene such that all mesenchyme-derived renal epithelial cells, from the Bowman's capsule to the junction of the connecting segment and collecting duct, are heritably labelled [18]. In contrast, the entire interstitial compartment is unlabelled [18]. Thus, the maintenance of labelled tubules post-injury would support a model of epithelial tubule repair due to surviving tubular epithelial cells or stem/progenitor cells localized within the labelled nephron, while label dilution would implicate an unlabelled, interstitial stem cell in the repair process. These findings indicate that papillary interstitial cells [14] or other types of interstitial stem/progenitor cells [8,9] do not directly contribute to renal epithelial cells regeneration. These observations also indicate that repair of injured nephrons is predominantly accomplished by intrinsic, surviving tubular epithelial cells or a subset of stem/progenitor cells localized within the nephron [18].

**Figure 1:**
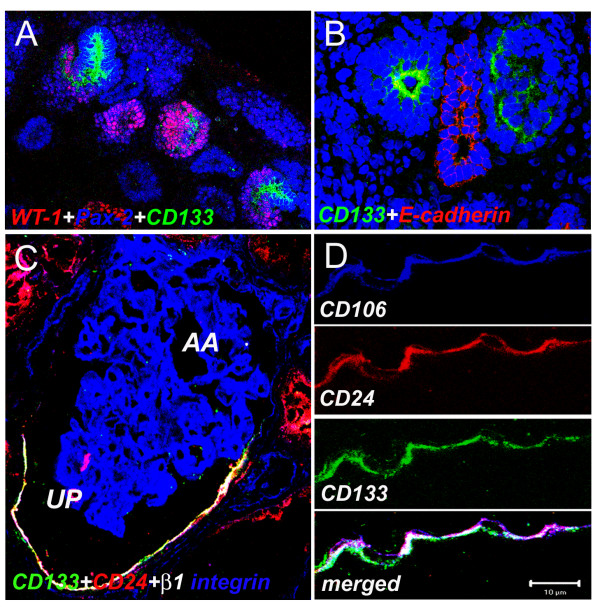
**CD24^+^CD133^+ ^renal progenitors localize at the urinary pole of the Bowman's capsule in adult human kidneys**. (A) Triple-label immunofluorescence for CD133, (green), CD24 (red) and β1 integrin (blue) showing that in a mature glomerulus, co-expression of CD133 and CD24 characterizes a subset of cells in the Bowman's capsule (white) localized at the urinary pole (UP). AA = afferent arteriola. Objective 20×. (B) High power magnification of a triple-label immunofluorescence for CD133, (green), CD24 (red) and CD106 (blue) in a subset of cells in the Bowman's capsule (white). Sections were stained as previously reported [15].

**Figure 2:**
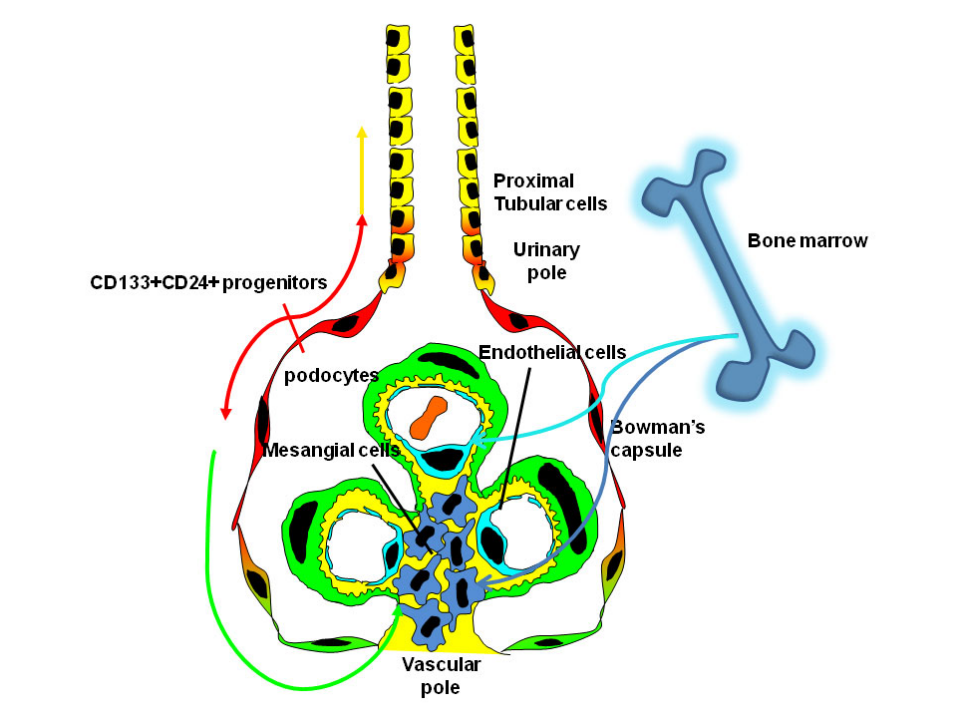
**Hypothetical diagram for kidney regeneration by different types of renal and extrarenal progenitors**. CD24+CD133+ renal progenitors (red) are localized at the urinary pole and are in close contiguity with podocytes (green) at one extremity (the vascular stalk) and with tubular renal cells (yellow) at the other extremity. A transitional cell population (red/green) displays features of either renal progenitors (red) or podocytes (green) and localizes between the urinary pole and the vascular pole. At the vascular stalk of the glomerulus, the transitional cells are localized in close continuity with cells that lack progenitor markers, but exhibit the podocyte markers and the phenotypic features of differentiated podocytes (green). On the opposite side, at the urinary pole, transitional cells (red/yellow) with a mixed phenotype between tubular cells (yellow) and progenitor cells (red). The directions of differentiation is indicated by the arrows (modified from [24]).

Recent studies identified a subset of renal stem/progenitor cells within the Bowman's capsule of adult human kidney [15]. These renal progenitors were identified through the assessment of the presence of both CD24, a surface molecule that has been used to identify different types of human stem cells [19,20] and CD133, a marker of several types of adult tissue stem cells [21,22]. The results showed that both markers were co-expressed by a subset of parietal epithelial cells selectively localized at the urinary pole of the Bowman's capsule [15] (Figure 1). Once isolated, CD24^+^CD133^+ ^renal progenitors were found to lack lineage-specific markers; to express transcription factors that are characteristic of multipotent stem cells, and to exhibit self-renewal, high clonogenic efficiency, and multidifferentiation potential [15]. When injected intravenously in SCID mice that had acute renal failure (ARF), CD24^+^CD133^+ ^renal progenitors regenerated tubular structures in different portions of the nephron and also reduced the morphological and functional kidney damage [15]. The identification of CD24^+^CD133^+ ^renal progenitors is in agreement with results obtained in transgenic mice, which suggests that endogenous cells of the nephron are responsible for repair of injured tubular epithelium [18], and allows the hypothesis that, from the urinary pole of the Bowman's capsule, CD24^+^CD133^+ ^renal progenitors might initiate the replacement and regeneration of tubular epithelial cells in adult human kidney [15] (Figure 2).

### Is there a unique renal progenitor common to renal development and the repair of adult kidney?

Regenerative biology draws on the understanding of normal developmental processes. It is generally believed that adult stem/progenitor cells represent a residual population directly derived from the organ-specific embryonic progenitor that is involved in organogenesis during fetal life [23-25]. This prompted us to evaluate whether co-expression of CD133 and CD24 might be useful to track down multipotent kidney stem/progenitor cells during human embryonic life.

Nephrons, the basic functional units of the kidney, are generated repetitively during kidney organogenesis from a mesenchymal progenitor population. Development of the mature mammalian kidney results from reciprocal signalling between the branching UB tips and the undifferentiated MM. This leads to the aggregation and condensation of renal epithelia progenitor cells to form the renal vesicle, which then undergoes transformation in the S-shaped body [2]. At this stage, the proximal end of the S-shaped body becomes invaded by blood vessels, differentiates into podocytes and parietal epithelial cells, and then generates glomeruli. Simultaneously, the middle and the distal segments of the S-shaped body begin to express proteins that are characteristic of tubular epithelia [2]. The existence of renal embryonic progenitors in the MM is supported by the observation that some MM-derived cells display multidifferentiation potential [2,26-28]. Accordingly, in embryonic human kidneys, co-expression of CD133 and CD24 characterizes a subset of cells in the cap mesenchyme, renal vesicles and S-shaped bodies that display self-renewal and multidifferentiation potential (Figure 1).

Interestingly, during nephron development, co-expression of CD133 and CD24 remained selectively localized to cells of the urinary pole of the Bowman's capsule [28]. Accordingly, CD24^+^CD133^+ ^renal embryonic progenitors progressively decreased during gestation and represented < 2% of whole cells in adult kidneys [15,28]. When injected into mice with ARF, CD24^+^CD133^+ ^renal embryonic progenitors regenerated cells of different portions of the nephron, reduced tissue necrosis and fibrosis, and significantly improved renal function [28]. In agreement with the putative nature of stem/progenitor cells, CD24^+^CD133^+ ^renal embryonic progenitors expressed high levels of the stem cell-specific transcription factor BmI-1 [29,30] and could generate tubular cells of different portions of the nephron in a model of acute tubular necrosis [28]. The existence of a putative MM cell with stem cell properties was already suggested by studies performed by the group of Reisner [31], who demonstrated that functioning renal tissue can be reconstituted by MM derived from kidneys of 8 weeks of gestation [31]. Indeed, fetal kidney tissue obtained from 10 to 14 weeks of gestation maintains the property to generate *de novo *functional nephrons, but generates a smaller number of mature glomeruli and tubuli than kidneys of 8 weeks of gestation [31]. Accordingly, CD24^+^CD133^+ ^renal embryonic progenitors are enriched in kidneys of 8 to 9 weeks of gestation, substantially decrease during 10 to 14 weeks of gestation, and represent < 2% of whole renal cells in adults.

It is interesting that in both fetal and adult kidney, CD24^+^CD133^+ ^progenitors persist as parietal epithelial cells localized at the urinary pole of the Bowman's capsule, supporting the concept that CD24^+^CD133^+ ^progenitors might represent a subpopulation of renal embryonic progenitors preserved from the early stages of nephrogenesis, and the urinary pole of the Bowman's capsule may represent stem cell niche, which is a specific site in adult tissues where stem cells reside (Figure 1) [32]. In agreement with this hypothesis, embryonic stem cells, once differentiated toward renal tubular cells, selectively migrated to the tubuloglomerular junction after injection into developing kidneys [17]. More recently, studies performed in transgenic rodents for the homeodomain transcriptional regulator Six2 have confirmed the existence in the cap mesenchyme of a multipotent nephron progenitor population [33]. Indeed, *Six2*-expressing cells give rise to all cell types of the main body of the nephron during all stages of nephrogenesis [34]. Pulse labeling of *Six2*-expressing nephron progenitors at the onset of kidney development suggests that the *Six2*-expressing population is maintained by self-renewal and is multipotent, generating the multiple domains of the whole cortical nephron [34]. Notably, descendants of a *Six2*+ cell can be found within molecularly distinct compartments of a single nephron – podocytes, proximal and distal tubule structures – further confirming that a single multipotent progenitor is the source of both the glomerular and tubular epithelial cells that constitute the adult nephron [34].

### Do bone marrow-derived cells contribute to tissue repair?

Some studies have suggested that cells from bone marrow might possess a surprising degree of plasticity and could differentiate into cell types of multiple organs of the body [33,35-38]. Accordingly, it was claimed that bone marrow-derived stem cells (BMSC) could contribute to the generation of new epithelial cells in functionally important numbers after kidney injury [39,40]. In light of their ease of accessibility, BMSC seem to be a very strong candidate for the treatment of renal diseases. Several subsequent studies have examined this possibility, with contrasting results [40-60].

Sugimoto and colleagues demonstrated that in a mouse model for Alport syndrome, bone marrow cells contribute to the emergence of viable podocytes which are associated with the production of new basement membrane [47]. In addition, unfractionated BMSC can differentiate into endothelial and mesangial cells in a model of progressive glomerulosclerosis [42,44] and, more surprisingly, they can form new tubular epithelial cells in functionally important numbers after kidney injury [43]. It has recently become clear that BMSC might fuse with differentiated cells in various adult organs, further complicating the interpretation of marrow transplantation studies [61]. Held and co-workers have shown that cell fusion could be induced between bone marrow-derived cells and renal tubular cells under conditions of chronic renal damage [60], apparently without impairment of cell division or conferment of genetic instability [44,62]. Additional works by several groups have shown that tubular cell replacement with BMSC is much lower than originally reported, calling into question the concept that BMSC physiologically participate in the repair of kidney injury [47-57]. Importantly, the low rate of functional improvement observed when using unfractionated BMSC suggests that in acute tubular injury, regenerating cells originated from intrarenal cells [6,7]. Accordingly, the absence of label dilution in Six2-GFPCre mice after injury and repair confirms that bone marrow-derived cells do not directly contribute to repair of tubular epithelial cells [18]. Taken together, these findings provide strong evidence against a physiological role for BMSC-derived cells in regeneration of post-ischaemic tubules by direct replacement of epithelial cells.

However, several studies indicate that mesangial cells might originate from a component of the hematopoietic lineages [62-65], and that BMSC might largely contribute to the regeneration of mesangial cells. Imasawa *et al*. [44] demonstrated the involvement of bone marrow-derived cells in normal mesangial cell turnover. Lethally irradiated mice given transplants of T-cell-depleted bone marrow cells from syngeneic donor transgenic for GFP manifested a time-dependent increase in GFP-positive cells in their glomeruli. When isolated and cultured, these cells stained positive for the mesangial cell marker desmin and the cells contracted in response to angiotensin II (Ang II), confirming that bone marrow-derived cells have the potential to differentiate into glomerular mesangial cells. Similar experiments with mice transplanted with purified clonally expanded hematopoietic progenitor cells were carried out by Masuya *et al*. [62] to confirm the hematopoietic origin of bone marrow-derived mesangial cells.

Finally, several studies have provided evidence that circulating endothelial progenitor cells (EPC) may contribute to glomerular capillary repair. In rat hematopoietic chimeras, low levels of bone marrow-derived cells staining for the rat endothelial cell antigen RECA-1 [66] were observed and the number of these cells gradually increased over time, suggesting that EPC contribute to normal physiological glomerular endothelial cell turnover. Following anti-Thy-1.1-induced glomerular injury the authors observed a fourfold increase in bone marrow-derived endothelial cells in the glomeruli [66]. These data indicate that glomerular repair cannot only be attributed to migration and proliferation of resident endothelial cells but it also involves bone marrow-derived cells.

Participation of circulating EPC in renal regeneration has also been demonstrated in human adults. Williams and Alvarez [67] were the first to describe the presence of acceptor endothelial cells in kidney allografts. Lagaaij *et al*. [68] reported that in human renal transplants the extent of replacement of donor endothelial cells lining the peritubular capillaries by those of the acceptor was related to the severity of vascular injury. They suggested that this endothelial replacement could be explained by the involvement of acceptor-derived EPC. Recently, male, donor-derived endothelial cells were observed in the renal macrovasculature of a female patient who developed thrombotic microangiopathy after gender-mismatched bone marrow transplantation [69]. Taken together, these observations confirm a role for BMSC in maintenance and repair of renal mesangium and endothelium, but not of the epithelial components of renal tissue.

### Towards the understanding of renal tissue regeneration

Tissue stem cells can form various lineages in response to physiological stimuli or injuries, a property that has great potential for regenerative medicine approaches. However, in many cases repair of epithelial cells does not depend on cells generated from multipotent stem cells, but directly derives from the migration of epithelial cells from the neighbouring epithelia, as previously reported also for the skin [70,71]. Indeed, genetic analyses suggest that the tubular epithelium can be self-renewing after acute kidney injury. Interestingly however, several previous studies have demonstrated that the proximal tubule arises at a variety of angles from Bowman's capsule and that at least one part of the tubuloglomerular junction has an area of intermediate appearance, with prominent microvilli on parietal cells in humans, mammals and fish. The finding of intermediate cells, especially in growing animals, suggests that parietal epithelium may be able to change to tubular and that this might particularly occur as the kidney grows, during severe renal disorders [72-75], following unilateral nephrectomy [76] or during ageing [77]. Thus, renal stem/progenitor cells might contribute to tubular epithelium repair, but this probably occurs only when a wound cannot spontaneously repair itself through the migration of neighbouring undamaged tubular cells (Figure 2).

Indeed, mice affected by rhabdomyolysis-induced acute tubular necrosis spontaneously recover from acute kidney injury, but mice undergoing early treatment with human renal progenitors show a complete recovery of renal function and kidney tissue integrity that was not observed in mice treated with saline [15] and, more importantly, a significant reduction of the severity of ARF, as revealed by the consistently lower blood urea nitrogen levels and extended areas of tubular tissue regenerated by human renal progenitors that co-expressed markers of proximal and distal tubules. This suggests that CD24^+^CD133^+ ^progenitors can regenerate tubular cells of different portions of the nephron, *in vitro *and *in vivo *[78,79]. However, the most important goal of regenerative medicine in the kidney is regeneration of glomerular injury, since glomerular diseases together account for 90% of end-stage kidney disease (ESKD).

Recent insights have defined a unified concept of glomerular diseases in which podocyte injury or loss is a common determining factor, which suggests the need for rational clinical efforts to allow podocyte regeneration [80-82]. Mature podocytes are post-mitotic cells, which can undergo DNA synthesis to a limited degree but do not proliferate, because they arrest in the G2/M phase of the cell cycle [80-82]. However, in most adult epithelia, replacement of damaged or dead cells is maintained through the presence of stem/progenitor cells [1]. Unless the epithelial stem/progenitor cells are permanently damaged, most epithelia are able to repair their tissues following injuries [1]. Although glomerular disorders represent the most prominent cause of ESKD, remission of the disease and regression of renal lesions have been observed in experimental animals and even in humans [3]. This shows that remodelling of glomerular architecture is possible, which would imply regeneration of the injured podocytes and reconstitution of the glomerular tuft. The inability of the podocyte to proliferate and replace injured cells suggests the existence of potential stem/progenitor cells within the adult glomerulus. Interestingly, CD24^+^CD133^+ ^renal progenitors are physically located within the Bowman's capsule, the only place in the kidney which appears to be contiguous with both tubular cells and glomerular podocytes [15]. Previous studies have suggested the existence of transitional cells exhibiting a mixed phenotype between the parietal epithelial cells and the podocyte at the vascular pole of the glomerulus [83]. In addition, CD24^+^CD133^+ ^renal progenitors represent common progenitors of tubular cells and podocytes during renal development [29]. Accordingly, recent studies performed in our laboratory suggest that CD24^+^CD133^+ ^renal progenitors can also regenerate glomerular podocytes in mice affected by adriamycin nephropathy, and can reduce the severity of proteinuria and of glomerular injury [84]. These results suggest that CD24^+^CD133^+ ^renal progenitors can also replace and regenerate podocytes through their division and migration along the Bowman's capsule towards the glomerular tuft during adult life or in response to podocyte injury (Figure 2).

### The response to renal injury: from regeneration to fibrosis

In humans, problems with wound healing can manifest as either delayed wound healing (which occurs with diabetes or radiation exposure) or excessive healing (as occurs with hypertrophic and keloid scars). Excessive healing is characterized by the deposition of large amounts of extracellular matrix and by alterations in local vascularization and cell proliferation. These excessive fibrotic reactions manifest in humans as a 'bad scar'. These commonly occur after major injuries such as burns, in which case they are referred to as hypertrophic scars. They can also appear for unknown reasons after a relatively minor trauma, as is the case for keloid scars, which might have a genetic basis [85-87]. Chronic infections, toxic and metabolic injuries, and idiopathic inflammatory diseases can promote the development of a scar, leading to tissue fibrosis [85]. In many cases, patients with progressive fibrosis have a poor prognosis and often require organ transplantation [85-87].

Although fibrosis is a part of the normal pathophysiological response to injury in many tissues, extended exposure to chronic injury results in tissue fibrosis, massive deposition of extracellular matrix, scar formation, and organ failure [87]. Chronic injury causes dysfunction of the tubular epithelial cells, which triggers release of fibrogenic cytokines and recruitment of inflammatory cells to injured kidneys [88-91]. Over the years, the primary focus of tubulointerstitial fibrosis studies has been on interstitial fibroblasts and infiltrated mononuclear cells for obvious reasons [92-94]. However, fibroblast activation after injury is, in essence, a wound-healing response by which the injured kidney attempts to repair and recover from the injury. Therefore, fibroblast activation at most may be necessary, but certainly not sufficient, for development of a full-scale of renal interstitial fibrosis.

**Figure 3:**
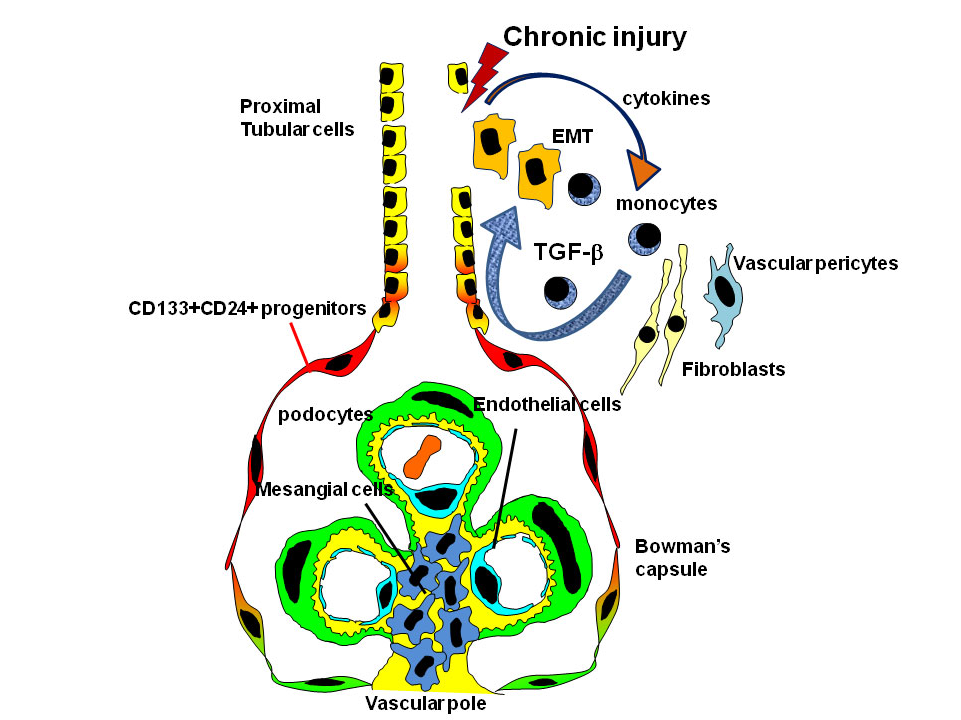
**Hypothetical diagram for kidney fibrosis**. Chronic injury causes dysfunction of the tubular epithelial cells, which triggers release of fibrogenic cytokines and recruitment of inflammatory cells to injured kidneys. Myelo-monocytic cells recruited from the bone marrow produce TGF-β1 in injured kidneys. In turn, TGF-β1 induces activation of collagen-producing cells, which mostly arise from kidney resident cells through epithelial-mesenchymal transition (modified [24]).

Fibroblasts contribute to 50% of all collagen-expressing cells in the course of renal fibrosis. Renal cortical fibroblasts maintain a quiescent state in normal kidneys, but in response to injury they proliferate and activate into myofibroblasts. Fibroblasts are not particularly abundant in normal kidneys as they are in lungs, lymph nodes, and spleen. When renal fibrogenesis sets in, about 36% of new fibroblasts come from the local epithelial mesenchymal transition (EMT), about 14–15% from the bone marrow, and the rest from local proliferation [94]. Endothelial cells also contribute to the emergence of fibroblasts during kidney fibrosis *via *the process of EMT, as recently demonstrated in mouse models of unilateral ureteral obstructive nephropathy, diabetic nephropathy, and Alport renal disease [95]. Although local activation of the renin-angiotensin system (RAS) and specifically Ang II affects all parenchymal organs, its effect is more pronounced in renal fibrosis. RAS stimulates inflammation, including the expression of cytokines, chemokines, growth factors, and reactive oxygen species [94,96]. Ang II induces vascular inflammation, endothelial dysfunction, up-regulation of adhesion molecules, and recruitment of infiltrating cells into the kidney (Figure 3) [94,96].

In addition, myelo-monocytic cells recruited from the bone marrow produce TGF-β1 in injured kidneys. In turn, TGF-β1 induces activation of collagen-producing cells, which mostly arise from kidney resident cells. The potential role of tubular epithelial cells in renal fibrosis is often concealed, partly because no direct connection seems to exist between tubular cells and the production and deposition of extracellular matrix, a hallmark of interstitial fibrosis. However, molecular analyses of gene expression have constantly underlined the potential importance of tubular epithelia in the fibrotic process. For instance, while it is well known that TGF-β1 expression is increased in almost all of the chronic kidney disease models studied, the expression of TGF-β receptors, which determine the specificity of TGF-β action, is often up-regulated predominantly in renal tubular epithelium [97], indicating that tubular epithelial cells are the *in vivo *natural targets of this pro-fibrotic cytokine. Hence, EMT helps to reconcile the disparity between molecular analysis and pathological findings in fibrotic kidney. Recently, pericytes were also identified as a major source of interstitial myofibroblasts in the fibrotic kidney, suggesting that either vascular injury or vascular factors are the most likely triggers for pericyte migration and differentiation into myofibroblasts (Figure 3) [98].

The accumulation of fibroblasts and an excess of collagen and other matrix components at sites of chronic inflammation lead to scar tissue formation and progressive tissue injury. These fibroblasts derive from the bone marrow, but also arise from an EMT of cells at injury sites [88,89]. EMT is likely to be involved in the progressive fibrotic diseases of the heart, lung, liver, and kidney, and genetic models provide indisputable evidence for a crucial role for EMT in renal fibrogenesis.

## Conclusion

Reversal of renal fibrosis is possible, as observed in experimental animals and even in humans [4]. However, whether fibrotic kidneys can reverse to normal renal architecture remains unresolved, and the point of no return in the development of irreversible renal fibrosis still remains to be determined. The rapid interposition of scar tissue probably confers a survival advantage by preventing infectious microorganisms from invading the wound and by inhibiting the continued mechanical deformation of larger tissues (a process that could compound the initial insult), but prevents subsequent tissue regeneration. However, the existence of renal epithelial progenitors in the kidney suggests a possible explanation for the regression of renal lesions, and indicates that a manipulation of the wound repair process in order to shift it towards regeneration [99] will probably require the ability to slow the rapid fibrotic response so that renal progenitor cells can regenerate functional tissue and avoid scar formation.

## Abbreviations

Ang II: angiotensin II; ARF: acute renal failure; BMSC: bone marrow-derived stem cells; BrdU: bromodeoxyuridine; EMT: epithelial mesenchymal transition; EPC: endothelial progenitor cells; ESKD: end stage kidney disease; GFP: green fluorescent protein; MM: metanephric mesenchyme; RAS: renin-angiotensin system; UB: ureteric bud

## Competing interests

The authors declare that they have no competing interests.

## Acknowledgements

The research leading to these results has received funding from the European Research Council Starting Grant under the European Community's Seventh Framework Programme (FP7/2007-2013), ERC grant number 205027. This study was also supported by the Tuscany Ministry of Health and the Associazione Italiana per la Ricerca sul Cancro.

## References

[B1] Blanpain C, Horsley V, Fuchs E (2007). Epithelial stem cells: turning over new leaves. Cell.

[B2] Dressler GR (2006). The cellular basis of kidney development. Annu Rev Cell Dev Biol.

[B3] Little MH (2006). Regrow or repair: potential regenerative therapies for the kidney. J Am Soc Nephrol.

[B4] Remuzzi G, Benigni A, Remuzzi A (2006). Mechanisms of progression and regression of renal lesions of chronic nephropathies and diabetes. J Clin Invest.

[B5] Imai E, Iwatani H (2007). The continuing story of renal repair with stem cells. J Am Soc Nephrol.

[B6] Lin F, Moran A, Igarashi P (2005). Intrarenal cells, not bone marrow-derived cells, are the major source for regeneration in post ischemic kidney. J Clin Invest.

[B7] Duffield JS, Park KM, Hsiao LL, Kelley VR, Scadden DT, Ichimura T, Bonventre JV (2005). Restoration of tubular epithelial cells during repair of the postischemic kidney occurs independently of bone marrow-derived stem cells. J Clin Invest.

[B8] Bussolati B, Bruno S, Grange C, Buttiglieri S, Deregibus MC, Cantino D, Camussi G (2005). Isolation of renal progenitor cells from adult human kidney. Am J Pathol.

[B9] Dekel B, Zangi L, Shezen E, Reich-Zeliger S, Eventov-Friedman S, Katchman H, Jacob-Hirsch J, Amariglio N, Rechavi G, Margalit R, Reisner Y (2006). Isolation and characterization of nontubular sca-1+lin_multipotent stem/progenitor cells from adult mouse kidney. J Am Soc Nephrol.

[B10] Gupta S, Verfaillie C, Chmielewski D, Kren S, Eidman K, Connaire J, Heremans Y, Lund T, Blackstad M, Jiang Y, Luttun A, Rosenberg ME (2006). Isolation and characterization of kidney-derived stem cells. J Am Soc Nephrol.

[B11] Kitamura S, Yamasaki Y, Kinomura M, Sugaya T, Sugiyama H, Maeshima Y, Makino H (2005). Establishment and characterization of renal progenitor like cells from S3 segment of nephron in rat adult kidney. FASEB J.

[B12] Maeshima A, Yamashita S, Nojima Y (2003). Identification of renal progenitor-like tubular cells that participate in the regeneration processes of the kidney. J Am Soc Nephrol.

[B13] Maeshima A, Sakurai H, Nigam SK (2006). Adult kidney tubular cell population showing phenotypic plasticity, tubulogenic capacity, and integration capability into developing kidney. J Am Soc Nephrol.

[B14] Oliver JA, Maarouf O, Cheema FH, Martens TP, Al-Awqati Q (2004). The renal papilla is a niche for adult kidney stem cells. J Clin Invest.

[B15] Sagrinati C, Netti GS, Mazzinghi B, Lazzeri E, Liotta F, Frosali F, Ronconi E, Meini C, Gacci M, Squecco R, Carini M, Gesualdo L, Francini F, Maggi E, Annunziato F, Lasagni L, Serio M, Romagnani S, Romagnani P (2006). Isolation and characterization of multipotent progenitor cells from the Bowman's capsule of adult human kidneys. J Am Soc Nephrol.

[B16] Kiel MJ, He S, Ashkenazi R, Gentry SN, Teta M, Kushner JA, Jackson TL, Morrison SJ (2007). Haematopoietic stem cells do not asymmetrically segregate chromosomes or retain BrdU. Nature.

[B17] Kim D, Dressler GR (2005). Nephrogenic factors promote differentiation of mouse embryonic stem cells into renal epithelia. J Am Soc Nephrol.

[B18] Humphreys BD, Valerius MT, Kobayashi A, Mugford JW, Soeung S, Duffield JS, McMahon AP, Bonventre JV (2008). Intrinsic epithelial cells repair the kidney after injury. Cell Stem Cell.

[B19] Shackleton M, Vaillant F, Simpson KJ, Stingl J, Smyth GK, Asselin-Labat ML, Wu L, Lindeman GJ, Visvader JE (2006). Generation of a functional mammary gland from a single stem cell. Nature.

[B20] Kubota H, Avarbock MR, Brinster RL (2003). Spermatogonial stem cells share some, but not all, phenotypic and functional characteristics with other stem cells. Proc Natl Acad Sci USA.

[B21] Coskun V, Wu H, Blanchi B, Tsao S, Kim K, Zhao J, Biancotti JC, Hutnick L, Krueger RC, Fan G, de Vellis J, Sun YE (2008). CD133+ neural stem cells in the ependyma of mammalian postnatal forebrain. Proc Natl Acad Sci USA.

[B22] Shmelkov SV, St Clair R, Lyden D, Rafii S (2005). AC133/CD133/Prominin-1. Int J Biochem Cell Biol.

[B23] Temple S (2001). The development of neural stem cells. Nature.

[B24] Morrison SJ, Kimble J (2006). Asymmetric and symmetric stem-cell divisions in development and cancer. Nature.

[B25] Self M, Lagutin OV, Bowling B, Hendrix J, Cai Y, Dressler GR, Oliver G (2006). Six2 is required for suppression of nephrogenesis and progenitor renewal in the developing kidney. EMBO J.

[B26] Oliver JA, Barasch J, Yang J, Herzlinger D, Al-Awqati Q (2002). Metanephric mesenchyme contains embryonic renal stem cells. Am J Physiol Renal Physiol.

[B27] Herzlinger D, Koseki C, Mikawa T, Al-Awqati Q (1992). Metanephric mesenchyme contains multipotent stem cells whose fate is restricted after induction. Development.

[B28] Lazzeri E, Crescioli C, Ronconi E, Mazzinghi B, Sagrinati C, Netti GS, Angelotti ML, Parente E, Ballerini L, Cosmi L, Maggi L, Gesualdo L, Rotondi M, Annunziato F, Maggi E, Lasagni L, Serio M, Romagnani S, Vannelli GB, Romagnani P (2007). Regenerative potential of embryonic renal multipotent progenitors in acute renal failure. J Am Soc Nephrol.

[B29] Molofsky AV, Pardal R, Iwashita T, Park IK, Clarke MF, Morrison SJ (2003). BmI-1 dependence distinguishes neural stem cell self-renewal from progenitor proliferation. Nature.

[B30] Kozakowski N, Soleiman A, Pammer J (2008). BMI-1 expression is inversely correlated with the grading of renal clear cell carcinoma. Pathol Oncol Res.

[B31] Dekel B, Burakova T, Arditti FD, Reich-Zeliger S, Milstein O, Aviel-Ronen S, Rechavi G, Friedman N, Kaminski N, Passwell JH, Reisner Y (2003). Human and porcine early kidney precursors as a new source for transplantation. Nat Med.

[B32] Scadden DT (2006). The stem-cell niche as an entity of action. Nature.

[B33] Krause DS, Theise ND, Collector MI, Henegariu O, Hwang S, Gardner R, Neutzel S, Sharkis SJ (2001). Multi-organ, multi-lineage engraftment by a single bone marrow-derived stem cell. Cell.

[B34] Kobayashi A, Valerius MT, Mugford JW, Carroll TJ, Self M, Oliver G, McMahon AP (2008). Six2 defines and regulates a multipotent self-renewing nephron progenitor population throughout mammalian kidney development. Cell Stem Cell.

[B35] Phinney DG, Prockop DJ (2007). Concise review: mesenchymal stem/multipotent stromal cells: the state of transdifferentiation and modes of tissue repair – current views. Stem Cells.

[B36] Spyridonidis A, Zeiser R, Follo M, Metaxas Y, Finke J (2005). Stem cell plasticity: the debate begins to clarify. Stem Cell Rev.

[B37] Lagasse E, Connors H, Al-Dhalimy M, Reitsma M, Dohse M, Osborne L, Wang X, Finegold M, Weissman IL, Grompe M (2000). Purified hematopoietic stem cells can differentiate into hepatocytes *in vivo*. Nat Med.

[B38] Herzog EL, Chai L, Krause DS (2003). Plasticity of marrow-derived stem cells. Blood.

[B39] Kale S, Karihaloo A, Clark PR, Kashgarian M, Krause DS, Cantley LG (2003). Bone marrow stem cells contribute to repair of the ischemically injured renal tubule. J Clin Invest.

[B40] Poulsom R, Forbes SJ, Hodivala-Dilke K, Ryan E, Wyles S, Navaratnarasah S, Jeffery R, Hunt T, Alison M, Cook T, Pusey C, Wright NA (2001). Bone marrow contributes to renal parenchymal turnover and regeneration. J Pathol.

[B41] Morigi M, Benigni A, Remuzzi G, Imberti B (2006). The regenerative potential of stem cells in acute renal failure. Cell Transplant.

[B42] Ikarashi K, Li B, Suwa M, Kawamura K, Morioka T, Yao J, Khan F, Uchiyama M, Oite T (2005). Bone marrow cells contribute to regeneration of damaged glomerular endothelial cells. Kidney Int.

[B43] Lin F, Cordes K, Li L, Hood L, Couser WG, Shankland SJ, Igarashi P (2003). Hematopoietic stem cells contribute to the regeneration of renal tubules after renal ischemia-reperfusion injury in mice. J Am Soc Nephrol.

[B44] Imasawa T, Utsunomiya Y, Kawamura T, Zhong Y, Nagasawa R, Okabe M, Maruyama N, Hosoya T, Ohno T (2001). The potential of bone marrow-derived cells to differentiate to glomerular mesangial cells. J Am Soc Nephrol.

[B45] Kunter U, Rong S, Boor P, Eitner F, Müller-Newen G, Djuric Z, van Roeyen CR, Konieczny A, Ostendorf T, Villa L, Milovanceva-Popovska M, Kerjaschki D, Floege J (2006). Transplanted mesenchymal stem cells accelerate glomerular healing in experimental glomerulonephritis. J Am Soc Nephrol.

[B46] Bi B, Schmitt R, Israilova M, Nishio H, Cantley LG (2007). Stromal cells protect against acute tubular injury via an endocrine effect. J Am Soc Nephrol.

[B47] Sugimoto H, Mundel TM, Sund M, Xie L, Cosgrove D, Kalluri R (2006). Bone-marrow-derived stem cells repair basement membrane collagen defects and reverse genetic kidney disease. Proc Natl Acad Sci USA.

[B48] Morigi M, Imberti B, Zoja C, Corna D, Tomasoni S, Abbate M, Rottoli D, Angioletti S, Benigni A, Perico N, Alison M, Remuzzi G (2004). Mesenchymal stem cells are renotropic, helping to repair the kidney and improve function in acute renal failure. J Am Soc Nephrol.

[B49] Tögel F, Hu Z, Weiss K, Isaac J, Lange C, Westenfelder C (2005). Administered mesenchymal stem cells protect against ischemic acute renal failure through differentiation-independent mechanisms. Am J Physiol Renal Physiol.

[B50] Lange C, Tögel F, Ittrich H, Clayton F, Nolte-Ernsting C, Zander AR, Westenfelder C (2005). Administered mesenchymal stem cells enhance recovery from ischemia/reperfusion-induced acute renal failure in rats. Kidney Int.

[B51] Tögel F, Weiss K, Yang Y, Hu Z, Zhang P, Westenfelder C (2007). Vasculotropic, paracrine actions of infused mesenchymal stem cells are important to the recovery from acute kidney injury. Am J Physiol Renal Physiol.

[B52] Herrera MB, Bussolati B, Bruno S, Morando L, Mauriello-Romanazzi G, Sanavio F, Stamenkovic I, Biancone L, Camussi G (2007). Exogenous mesenchymal stem cells localize to the kidney by means of CD44 following acute tubular injury. Kidney Int.

[B53] Li B, Morioka T, Uchiyama M, Oite T (2006). Bone marrow cell infusion ameliorates progressive glomerulosclerosis in an experimental rat model. Kidney Int.

[B54] Kunter U, Rong S, Boor P, Eitner F, Müller-Newen G, Djuric Z, van Roeyen CR, Konieczny A, Ostendorf T, Villa L, Milovanceva-Popovska M, Kerjaschki D, Floege J (2007). Mesenchymal stem cells prevent progressive experimental renal failure but maldifferentiate into glomerular adipocytes. J Am Soc Nephrol.

[B55] Imberti B, Morigi M, Tomasoni S, Rota C, Corna D, Longaretti L, Rottoli D, Valsecchi F, Benigni A, Wang J, Abbate M, Zoja C, Remuzzi G (2007). Insulin-like growth factor-1 sustains stem cell mediated renal repair. J Am Soc Nephrol.

[B56] Herrera MB, Bussolati B, Bruno S, Fonsato V, Romanazzi GM, Camussi G (2004). Mesenchymal stem cells contribute to the renal repair of acute tubular epithelial injury. Int J Mol Med.

[B57] Prodromidi EI, Poulsom R, Jeffery R, Roufosse CA, Pollard PJ, Pusey CD, Cook HT (2006). Bone marrow-derived cells contribute to podocyte regeneration and amelioration of renal disease in a mouse model of Alport syndrome. Stem Cells.

[B58] Ninichuk V, Gross O, Segerer S, Hoffmann R, Radomska E, Buchstaller A, Huss R, Akis N, Schlöndorff D, Anders HJ (2006). Multipotent mesenchymal stem cells reduce interstitial fibrosis but do not delay progression of chronic kidney disease in collagen4A3-deficient mice. Kidney Int.

[B59] Guo JK, Schedl A, Krause DS (2006). Bone marrow transplantation can attenuate the progression of mesangial sclerosis. Stem Cells.

[B60] Held PK, Al-Dhalimy M, Willenbring H, Akkari Y, Jiang S, Torimaru Y, Olson S, Fleming WH, Finegold M, Grompe M (2006). *In vivo *genetic selection of renal proximal tubules. Mol Ther.

[B61] Rizvi AZ, Swain JR, Davies PS, Bailey AS, Decker AD, Willenbring H, Grompe M, Fleming WH, Wong MH (2006). Bone marrow-derived cells fuse with normal and transformed intestinal stem cells. Proc Natl Acad Sci USA.

[B62] Masuya M, Drake CJ, Fleming PA, Reilly CM, Zeng H, Hill WD, Martin-Studdard A, Hess DC, Ogawa M (2003). Hematopoietic origin of glomerular mesangial cells. Blood.

[B63] Abe T, Fleming PA, Masuya M, Minamiguchi H, Ebihara Y, Drake CJ, Ogawaa M (2005). Granulocyte/macrophage origin of glomerular mesangial cells. Int J Hematol.

[B64] Takeda S, Rogers SA, Hammerman MR (2006). Differential origin for endothelial and mesangial cells after transplantation of pig fetal renal primordia into rats. Transpl Immunol.

[B65] Rookmaaker MB, Verhaar MC, Van Zonneveld AJ, Rabelink TJ (2004). Progenitor cells in the kidney: Biology and therapeutic perspectives. Kidney Int.

[B66] Rookmaaker MB, Tolboom H, Goldschmeding R, Zwaginga JJ, Rabelink TJ, Verhaar MC (2003). Bone marrow-derived cells contribute to glomerular endothelial repair in experimental glomerulonephritis. Am J Pathol.

[B67] Williams GM, Alvarez CA (1969). Host repopulation of the endothelium in allografts of kidneys and aorta. Surg Forum.

[B68] Lagaaij EL, Cramer-Knijnenburg GF, van Kemenade FJ, van Es LA, Bruijn JA, van Krieken JH (2001). Endothelial cell chimerism after renal transplantation and vascular rejection. Lancet.

[B69] Rookmaaker MB, Tolboom H, Goldschmeding R, Zwaginga JJ, Rabelink TJ, Verhaar MC (2002). Bone-marrow-derived cells contribute to endothelial repair after thrombotic microangiopathy. Blood.

[B70] Ito M, Liu Y, Yang Z, Nguyen J, Liang F, Morris RJ, Cotsarelis G (2005). Stem cells in the hair follicle bulge contribute to wound repair but not to homeostasis of the epidermis. Nature Med.

[B71] Levy V, Lindon C, Harfe BD, Morgan BA (2005). Distinct stem cell populations regenerate the follicle and interfollicular epidermis. Dev Cell.

[B72] Finckh ES, Joske RA (1954). The occurrence of columnar epithelium in Bowman's capsule. J Path Bact.

[B73] Nachman RL (1962). Metaplasia of parietal capsular epithelium of renal glomerulus. Arch Path.

[B74] Kanel GC, Peters RL (1984). Glomerular tubular reflux – a morphologic renal lesion associated with the hepatorenal syndrome. Hepatology.

[B75] Valdes AJ, Zhang JM (1987). Intraglomerular tubular epithelial cells. A marker of glomerular hematuria. Arch Pathol Lab Med.

[B76] Andrews PM (1981). The presence of proximal tubule-like cells in the kidney parietal epithelium in response to unilateral nephrectomy. Anat Rec.

[B77] Castelletto L, Goya RG (1990). Sex-related incidence of tubular metaplasia in Bowman's capsule of aging rats. Virchows Arch B Cell Pathol Incl Mol Pathol.

[B78] Mazzinghi B, Ronconi E, Lazzeri E, Sagrinati C, Ballerini L, Angelotti ML, Parente E, Mancina R, Netti GS, Becherucci F, Gacci M, Carini M, Gesualdo L, Rotondi M, Maggi E, Lasagni L, Serio M, Romagnani S, Romagnani P (2008). Essential but differential role for CXCR4 and CXCR7 in the therapeutic homing of human renal progenitor cells. J Exp Med.

[B79] Sagrinati C, Ronconi E, Lazzeri E, Lasagni L, Romagnani P (2008). Stem-cell approaches for kidney repair: choosing the right cells. Trends Mol Med.

[B80] Wiggins RC (2007). The spectrum of podocytopathies: a unifying view of glomerular diseases. Kidney Int.

[B81] D'Agati VD (2008). Podocyte injury in focal segmental glomerulosclerosis: Lessons from animal models (a play in five acts). Kidney Int.

[B82] Wharram BL, Goyal M, Wiggins JE, Sanden SK, Hussain S, Filipiak WE, Saunders TL, Dysko RC, Kohno K, Hozman LB, Wiggins RC (2005). Podocyte depletion causes glomerulosclerosis: diphtheria toxin-induced podocyte depletion in rats expressing human diphtheria toxin receptor transgene. J Am Soc Nephrol.

[B83] Bariety J, Mandet C, Hill GS, Bruneval P (2006). Parietal podocytes in normal human glomeruli. J Am Soc Nephrol.

[B84] Ronconi E, Sagrinati C, Angelotti ML, Lazzeri E, Mazzinghi B, Ballerini L, Parente E, Becherucci F, Gacci M, Carini M, Maggi E, Serio M, Vannelli GB, Lasagni L, Romagnani S, Romagnani P (2009). Regeneration of glomerular podocytes by human renal progenitors. J Am Soc Nephrol.

[B85] Gurtner GC, Werner S, Barrandon Y, Longaker MT (2008). Wound repair and regeneration. Nature.

[B86] Singer AJ, Clark RA (1999). Cutaneous wound healing. N Engl J Med.

[B87] Aarabi S, Longaker MT, Gurtner GC (2007). Hypertrophic scar formation following burns and trauma: new approaches to treatment. PLoS Med.

[B88] Neilson EG (2006). Mechanisms of disease: Fibroblasts – a new look at an old problem. Nat Clin Pract Nephrol.

[B89] Kalluri R, Neilson EG (2003). Epithelial-mesenchymal transition and its implications for fibrosis. J Clin Invest.

[B90] Ruiz-Ortega M, Rodriguez-Vita J, Sanchez-Lopez E, Carvajal G, Egido J (2007). TGF-beta signaling in vascular fibrosis. Cardiovasc Res.

[B91] Ruiz-Ortega M, Ruperez M, Esteban V, Rodriguez-Vita J, Sanchez-Lopez E, Carvajal G, Egido J (2006). Angiotensin II: a key factor in the inflammatory and fibrotic response in kidney diseases. Nephrol Dial Transplant.

[B92] Kuwano K, Hagimoto N, Kawasaki M, Yatomi T, Nakamura N, Nagata S, Suda T, Kunitake R, Maeyama T, Miyazaki H, Hara N (1999). Essential roles of the Fas-Fas ligand pathway in the development of pulmonary fibrosis. J Clin Invest.

[B93] Hinz B, Phan SH, Thannickal VJ, Galli A, Bochaton-Piallat ML, Gabbiani G (2007). The myofibroblast: one function, multiple origins. Am J Pathol.

[B94] Kisseleva T, Uchinami H, Feirt N, Quintana-Bustamante O, Segovia JC, Schwabe RF, Brenner DA (2006). Bone marrow-derived fibrocytes participate in pathogenesis of liver fibrosis. J Hepatol.

[B95] Zeisberg EM, Potenta SE, Sugimoto H, Zeisberg M, Kalluri R (2008). Fibroblasts in kidney fibrosis emerge via endothelial-to-mesenchymal transition. J Am Soc Nephrol.

[B96] Sironi L, Nobili E, Gianella A, Gelosa P, Tremoli E (2005). Anti-inflammatory properties of drugs acting on the renin-angiotensin system. Drugs Today (Barc).

[B97] Herzlinger D (2002). Renal interstitial fibrosis: remembrance of things past?. J Clin Invest.

[B98] Lin SL, Kisseleva T, Brenner DA, Duffield JS (2008). Pericytes and perivascular fibroblasts are the primary source of collagen-producing cells in obstructive fibrosis of the kidney. Am J Pathol.

[B99] Romagnani P, Lasagni L, Mazzinghi B, Lazzeri E, Romagnani S (2007). Pharmacological modulation of stem cell function. Curr Med Chem.

